# Coyote (*Canis latrans*) Macronutrient Consumption and Diet Relative to Seasonality and Urbanization

**DOI:** 10.1002/ece3.71405

**Published:** 2025-05-12

**Authors:** Katherine C. B. Weiss, Sean C. P. Coogan, Pierre Deviche, Jesse S. Lewis, Savage C. Hess, Jan Schipper, Eric G. Strauss, Beckett Sterner

**Affiliations:** ^1^ School of Life Sciences Arizona State University Tempe Arizona USA; ^2^ Department of Environmental Science & Policy Marist University Poughkeepsie New York USA; ^3^ Department of Renewable Resources University of Alberta Edmonton Alberta Canada; ^4^ Department of Natural Resource Science Thompson Rivers University Kamloops Canada; ^5^ College of Integrative Sciences and Arts Arizona State University Mesa Arizona USA; ^6^ The New College of Interdisciplinary Arts and Sciences Arizona State University – West Glendale Arizona USA; ^7^ Arizona Center for Nature Conservation/Phoenix Zoo Phoenix AZ USA; ^8^ Biology Department & Center for Urban Resilience Loyola Marymount University Los Angeles California USA

**Keywords:** Arizona, carnivore, coyote, diet selection, mammal, nutritional geometry, omnivore

## Abstract

Diet selection informs the health, fitness, and behavior of wild predators. Due to assumptions that vertebrate prey contains similar compositions of macronutrients (i.e., protein, carbohydrates, and lipids), whole prey items traditionally define carnivore diets. However, increasing evidence suggests that prey differ in terms of their macronutrient compositions, particularly relative to body size. Furthermore, omnivorous predators, like coyotes ( *Canis latrans* ), integrate both prey and nonprey diet items whose macronutrient compositions vary. This is particularly important in urbanized systems, which introduce or alter the distributions of prey (e.g., domestic pets) and nonprey (e.g., ornamental plants) foods in ways that contribute to carnivore diet selection and human–wildlife coexistence. We assessed the macronutrient composition of coyote diets seasonally and relative to urbanization in the Phoenix Metropolitan Area, AZ, USA. We collected coyote scats in the field and assessed their macronutrient compositions using values gathered from the literature, as well as the volumetric composition of diet items found in coyote scats. We then assessed the macronutrient composition of coyote diets in geometric space using the geometric framework of nutrition. We observed that the macronutrient composition of coyote diets was similar between moderately and less urbanized sites, particularly in the spring–summer season. However, coyote macronutrient consumption differed seasonally, with coyotes eating more nonprotein energy relative to protein energy when carbohydrate‐rich mesquite (*Prosopis* spp.) was more available in the fall–winter. Our results suggest that the seasonal availability and macronutrient composition of foods contribute to coyote diets. Macronutrients directly translate to energy and subsequent animal physiology and behavior. Our findings therefore advance our understanding of coyote behavior, particularly in ways that support human–wildlife management in anthropogenic areas.

## Graphical abstract

Using the geometric framework of nutrition, we assessed the macronutrient composition of coyote diets seasonally and relative to urbanization via scats collected in the Phoenix Metropolitan Area, AZ, USA. We found coyotes ate more nonprotein energy relative to protein energy when seasonally or environmentally available. Our results suggest that the seasonal availability and macronutrient composition of foods contribute to coyote diet selection.

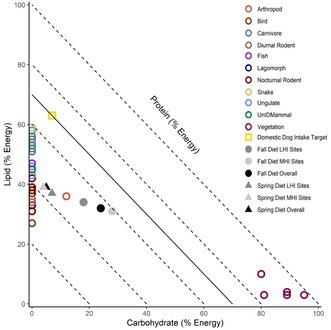

## 1 Introduction

Differential diet selection influences the fitness (Jensen et al. 2012), habitat selection (Johnson 1980), health (Murray, Edwards, et al. 2015; Sugden et al. 2020), and behavior (Young et al. 2019; West and Jones 2022) of wild predators. Traditional approaches describe predation based on the availability of whole food items within the environment as opposed to nutrition, since prey species are assumed to have similar nutrient compositions (Holling 1959a, 1959b; Wangersky 1978; Allen 1988; Kohl et al. 2015). However, this perspective does not consider how the macronutrient (i.e., protein, carbohydrate, and lipid) composition of prey varies with taxonomy and body size (Kohl et al. 2015; Machovsky‐Capuska, Coogan, et al. 2016), or how the diets of omnivorous predators change seasonally to incorporate nonprey taxa (Coogan, Raubenheimer, Stenhouse, et al. 2018; Jensen et al. 2022). These dynamics become more pronounced in urbanized areas, which introduce novel and supplemental food sources that alter trophic dynamics (Faeth et al. 2005; El‐Sabaawi 2018) and the nutritional environment (Jones and Reynolds 2008; Bateman and Fleming 2012; Wist et al. 2022; Brown et al. 2022). It is therefore likely that omnivorous carnivores forage for select nutrients (Kohl et al. 2015; Machovsky‐Capuska, Coogan, et al. 2016; Coogan, Raubenheimer, Stenhouse, et al. 2018) in anthropogenic environments.

Coyotes ( *Canis latrans* ) are a highly adaptable, omnivorous carnivore species and dietary generalist found throughout North America and most major cities in the USA and Canada (Hody and Kays 2018). Their diets include a wide range of vertebrate and invertebrate prey, such as rabbits, small rodents, birds, domestic pets, ungulates, herpetofauna, arthropods, and fish (Gehrt and Riley 2010; Jensen et al. 2022). Though coyotes primarily consume prey taxa that contain no or low proportions of carbohydrates (i.e., prey species), they also frequently eat fruits, seeds, trash, and human foods when available, either seasonally or geographically (Newsome et al. 2015; Larson et al. 2020; Jensen et al. 2022). Since macronutrient consumption can contribute to animal health, behavior, cognition, and reproduction (Birnie‐Gauvin et al. 2017), understanding coyote macronutrient selection is important for wildlife management in urbanized areas. For example, several studies have investigated differential protein consumption by coyotes, which has been associated with changes in trophic position, the microbiome (Colborn et al. 2020; Sugden et al. 2020), degrees of human–wildlife conflict (Murray, Cembrowski, et al. 2015), and the propensity for parasitic infections (Murray, Edwards, et al. 2015; Sugden et al. 2020). Though it is helpful to assess protein selection by coyotes, animals do not consume macronutrients in isolation. Instead, animals consume different ratios of macronutrients relative to what is available in the environment and given individual preferences and physiological requirements and constraints (Simpson and Raubenheimer 2012). In addition, since all food items contain different combinations of macronutrients, animals must make decisions about what they eat, not only depending on the local availability of whole foods but also given the nutritional composition of individual diet items (Kohl et al. 2015). Further investigation is therefore needed to understand the complete macronutrient intake (i.e., of proteins, carbohydrates, and lipids, collectively) by coyotes and whether this changes with urbanization.

Macronutrient intake regulation can affect animal lifespan (Jensen et al. 2012) reproduction, and health (Solon‐Biet et al. 2014), and nutritional ecology research has demonstrated that many animals regulate their nutritional consumption to achieve an “intake target,” or a preferred ratio of nutrients, that optimizes fitness (Simpson and Raubenheimer 2012). This intake target is analogous to a fundamental macronutrient niche (Simpson and Raubenheimer 2012; Machovsky‐Capuska, Senior, et al. 2016). However, in reality, wild animals are rarely able to consume their ideal ratio of macronutrients, due to the availability of foods seasonally and interannually, inter‐ and intraspecific competition, and other environmental characteristics (e.g., urbanization, climatic changes) (Machovsky‐Capuska, Senior, et al. 2016). When unable to reach their intake target, animals will alter their consumption of foods to best approximate this target by undereating some nutrients while overconsuming others (Simpson and Raubenheimer 2012). Many animals, including humans, regulate protein (i.e., protein prioritization) more than nonprotein macronutrients (i.e., carbohydrate and lipid) (Simpson and Raubenheimer 2005). For example, grizzly bears (*Ursus arctos horribillis*) and domestic dogs ( *Canis familiaris* ) control their protein intake by varying their consumption of nonprotein energy (Hewson‐Hughes et al. 2013; Coogan et al. 2014). This tight control of protein intake may have evolved as a result of protein overconsumption being associated with reduced lifespans in several model organisms (Raubenheimer et al. 2016), as well as negative health outcomes in some carnivores (e.g., Rode et al. 2021). Furthermore, the consumption of imbalanced diets has implications for wildlife species, which are subject to seasonal, interannual, spatial, and nutritional variability in food resources (Coogan et al. 2014; Coogan, Raubenheimer, Stenhouse, et al. 2018). Thus, discerning how macronutrient availability and consumption changes seasonally and relative to altered landscapes is important for understanding the health and longevity of coyotes.

Urbanization significantly alters the nutritional environment for wildlife (Birnie‐Gauvin et al. 2017), particularly in the availability of macronutrients (Coogan, Raubenheimer, Zantis, and Machovsky‐Capuska 2018; Stofberg et al. 2019; Brown et al. 2022). For example, human‐dominated landscapes increase the availability of carbohydrates (Murray, Cembrowski, et al. 2015; Stofberg et al. 2019; Wist et al. 2022) and lipids (e.g., processed foods; Townsend et al. 2019), as well as introduce non‐native protein and fat sources (e.g., domestic pets; Flockhart et al. 2016; Larson et al. 2020; Kays et al. 2020; Reed et al. 2023). Macronutrients directly translate to energy, and so the differential availability and/or consumption of macronutrients can have significant implications for organismal fitness via growth and reproduction (Kohl et al. 2015; Birnie‐Gauvin et al. 2017). Furthermore, urbanization influences the nutritional quality of foods, which can impact wildlife physiology and behavior by promoting stress (e.g., cortisol, oxidative stress, and inflammation), altering body condition, increasing rates of disease, as well as reducing the success of some management interventions (Ditchkoff et al. 2006; Murray, Edwards, et al. 2015; Murray et al. 2019; Isaksson 2015; Birnie‐Gauvin et al. 2017; Stothart et al. 2019; Young et al. 2019; Sugden et al. 2020; Bernat‐Ponce et al. 2023). Urbanization can also alter coyote occupancy (Gehrt and Riley 2010; Bateman and Fleming 2012; Hody and Kays 2018), diet (Gehrt and Riley 2010; Newsome et al. 2015; Larson et al. 2020), behavior (Breck et al. 2019), predator–prey relationships in both space and time (Weiss 2024), as well as interactions with people (Young et al. 2019). Understanding coyote macronutrient intake may therefore provide key information to promote more sustainable futures between people and coyotes.

We used scat and nutritional geometric approaches (Simpson and Raubenheimer 2012) to assess the macronutrient composition and volumetric contents of coyote diets seasonally and across a gradient of urbanization along the Salt River corridor in the Phoenix Metropolitan Area, AZ, USA. We hypothesized that coyote macronutrient consumption would change with seasonal availability, similar to other omnivorous carnivores (e.g., Coogan et al. 2014). Specifically, we predicted that coyotes would consume more nonprotein energy when seasonally available, as seen in other carnivore species (e.g., Coogan et al. 2014; Rode et al. 2021). We also hypothesized that coyote macronutrient consumption would vary with the degree of urbanization, due to expected differences in macronutrient availability between areas of low and moderate human influence. When assuming protein needs were already met, as determined relative to the protein intake target of domestic dogs (Hewson‐Hughes et al. 2013), we expected coyotes in more urbanized sites to consume more nonprotein energy than coyotes in less urbanized sites.

## 2 Materials and Methods

### 2.1 Study Area

We conducted our study along the Salt River corridor in Maricopa County, AZ, USA across two seasons. Overall, this arid region is characterized by mild springs, falls, and winters with occasional rain, as well as hot summers interspersed with monsoon rains (Wang et al. 2018; US Climate Data 2023). We collected data across two seasons, between March–June 2021 (spring–summer season) and October 2021–January 2022 (fall–winter season). The average monthly temperature in the spring–summer season was 26.6°C (79.8°F), with an average minimum monthly temperature of 19.4°C (66.9°F) and an average monthly high temperature of 33.8°C (92.8°F) (NOAA 2021). Total precipitation in the spring–summer season averaged 0.36 cm (NOAA 2021). Meanwhile, the average monthly temperature in the fall–winter season was 18.6°C (65.5°F), with an average minimum monthly temperature of 11.9°C (53.5°F) and an average high monthly temperature of 25.1°C (77.2°F) (NOAA 2021, 2022). Total precipitation in the fall averaged 1.2 cm (NOAA 2021, 2022). This region is characterized by Sonoran Desert flora, including mesquite trees (*Prosopis* spp.), creosote bush ( *Larrea tridentata* ), saguaro ( *Carnegiea gigantea* ), cholla cacti (*Cylindropuntia* spp.), ocotillo ( *Fouquieria splendens* ), brittlebush (*Encelia farinose*), prickly pear (*Opuntia* spp.), and many other desert‐adapted plants (Turner et al. 1995). More urbanized stretches of this region also commonly maintain introduced palm trees (Arecaceae spp.) (Schuch and Quist 2023), native mesquite species, and many other introduced plant species (Martin et al. 2004).

### 2.2 Classifying Urbanization

**FIGURE 1:**
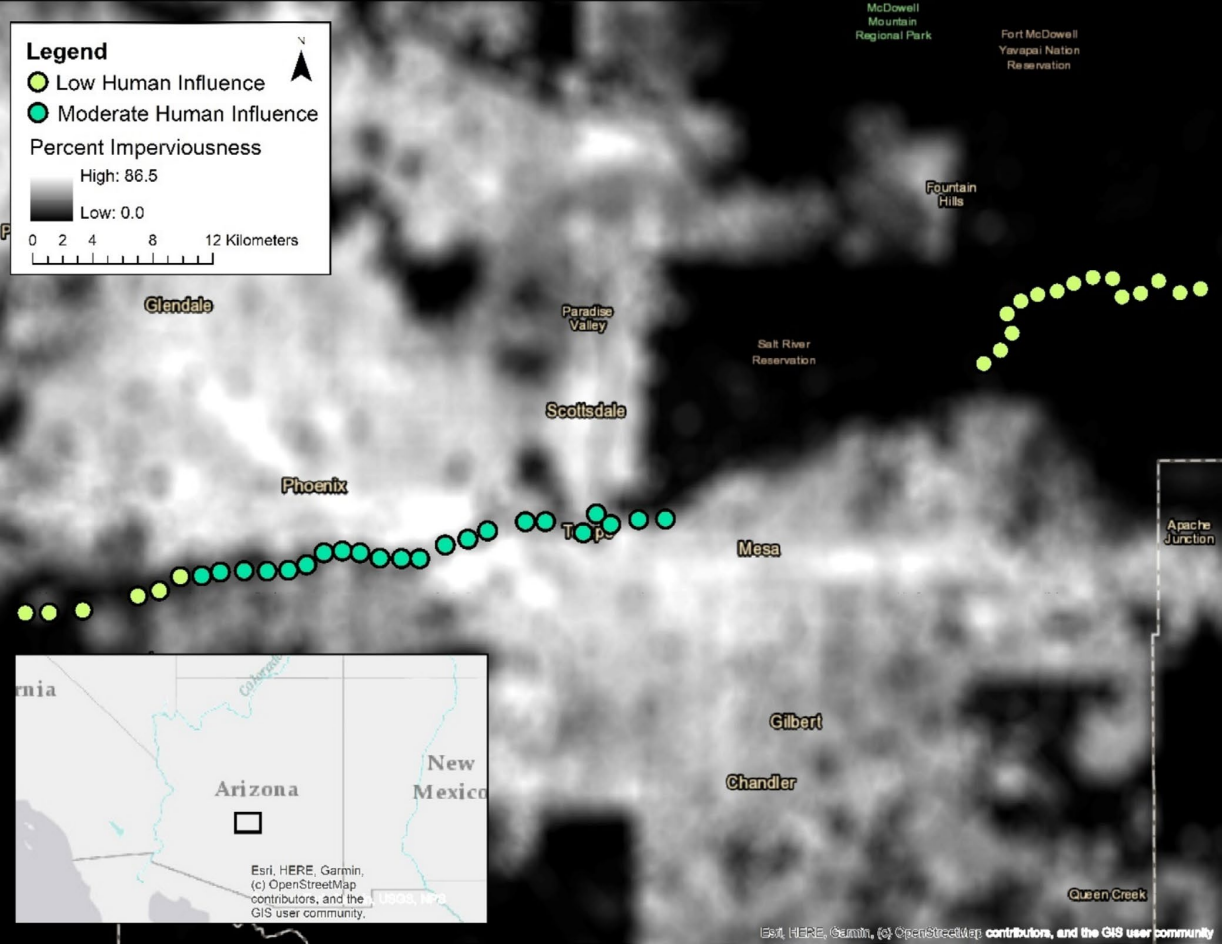
Locations of remote wildlife camera and coyote scat collection along the Salt River corridor in the Phoenix Metropolitan Area, AZ, USA across two seasons (spring–summer and fall–winter) and a gradient of urbanization. Low human influence (0%–20%) and moderate human influence (21%–60%) were determined using average National Landcover Database (NLCD) percent impervious surface cover data within 1000 m radius buffers of each site (Dewitz and US Geological Survey 2021).

We used National Landcover Database percent impervious surface cover data to categorize urbanization in our study region (Dewitz and US Geological Survey 2021). Using Google Earth Engine (Gorelick et al. 2017), we then created an impervious surface layer by calculating the average impervious cover within a 1000 m radius buffer of each 25 × 25 cell. We also investigated using 500 m radius buffers to categorize urbanization in our study region, though both 1000 and 500 m radius buffers were highly correlated (*r* = 0.94, *p* < 0.001). In addition, we sought to capture urbanization more broadly across the region and relative to the scale at which coyotes might perceive and interact with landscape characteristics. As such, we continued with using the layer of percent impervious surface cover within 1000 m radius buffers to determine the average impervious surface associated with each site using the “Extract Values to Points (Spatial Analyst)” tool in ArcMap 10.7.1 (Redlands, CA: Environmental Systems Research Institute Inc.). Using a histogram approach, we then classified sites into either low human influence (LHI) if the average impervious surface within a 1000 m radius buffer of a site was 0%–20% (*n* = 20, $\bar{x}$: 0.04 (SD: 0.05)) or moderate human influence (MHI) if the average impervious surface within a 1000 m buffer of a site was 21%–60% (*n* = 23, $\bar{x}$: 0.41 (SD: 0.12)) (Figure 1).

### 2.3 Scat Data Collection and Processing

Scats were collected and processed according to Hess et al. (2024). Briefly, scat data were collected monthly during the study duration based on morphology along 500 m transects. Transects were recorded using the Gaia GPS phone application (https://www.gaiagps.com/). All scats throughout each transect were cleared 1 month prior to collection in both seasons. We then visited each site monthly for 2 months to collect scats. As fewer scats were found in sites throughout the Tonto National Forest study area (eastern LHI sites in Figure 1), these sites were visited an additional month in each season to balance sample sizes. Scats were identified morphologically (Brown et al. 2020) and collected in plastic bags with associated spatial locations and percent confidence (50%–100%) in species identification by sight. Scats were then stored in a −80°C freezer. To confirm our identification of coyote scats was accurate, scats with < 100% confidence in species ID morphologically were swabbed for remnant epithelial DNA and sent for microsatellite marker genetic testing to confirm these scats were indeed deposited by a coyote (https://wildlifegenetics.ca/). A random subset of coyote scats with 100% confidence in morphological ID were also swabbed for DNA to ensure our ability to detect coyote scats by sight overall was accurate. Any scats determined from genetic analysis to not belong to a coyote or that had < 100% confidence in ID but were unable to be sent for genetic testing were removed from the study and not analyzed. To sanitize the scats prior to hard‐parts identification, we baked them in an oven for 24 h at 60°C (Brown et al. 2020). We then soaked scats in soapy water for 24–48 h and rinsed out any remaining fecal material using a cheese cloth to isolate the dietary remains left in the scats for identification. Scat contents were then left to air dry for a subsequent 24–48 h before storing in envelopes.

### 2.4 Food Item Identification

We classified dietary items as mammal (to the lowest taxonomic classification possible), bird, snake, fish, arthropod (including insects, scorpions, and crayfish), vegetation (to family or genus, when possible), trash, eggshell, and unidentifiable (including within each mammal category and overall). To identify mammals, five random samples of hair per scat were wet‐mounted and reviewed under a microscope (AmScope B120, United Scope LLC, Irvine, CA) (Morin et al. 2019) and photographed to compare with reference slides of hair using specimens from Arizona State University's Natural History Collections, identification keys (Moore et al. 1974; Debelica and Thies 2009; Larson 2020), and expert solicitation. We also identified the remains of teeth found in scats using reference images from specimens at Arizona State University's Natural History Collections, as well as expert solicitation. In addition to hair, domestic cat remains were identified via the presence of domestic cat claws and teeth. Domestic cats were further identified from bobcats ( *Lynx rufus* ) via hair color and texture, as well as location, when compared with where bobcats were detected on camera traps in an associated research project that used the same study sites in the same time period scats were collected (Weiss 2024). Similarly, domestic dogs ( *Canis familiaris* ) were identified from coyotes based on hair color and texture, as well as expert review. Lagomorph hairs were classified as desert cottontail rabbit ( *Sylvilagus audubonii* ) or unidentifiable Lagomorph based on the number of rows of medulla within a sample being either greater than four (i.e., cottontail rabbit) or less than or equal to four (i.e., unidentifiable between cottontail rabbit or black‐tailed jackrabbit, *Lepus californicus* ) (Debelica and Thies 2009). Any sample with at least one lagomorph hair greater than four medulla cells wide was determined to belong to a cottontail rabbit—and was often corroborated by lagomorph teeth of a smaller size. Lagomorph samples with only thinner hairs in terms of medullar width were deemed as belonging to an unidentifiable lagomorph. Neotominae species (i.e., *Neotoma* or *Peromyscus* spp.) were grouped together due to the similarity in their hair structure. Bird remains were identified via feathers or hollow bones present in the scats, while reptiles and fish were identified via scales and confirmed via expert review. Due to desiccation, crayfish remains were only identifiable when washing the scats prior to drying. Vegetation, particularly seeds, were identified via expert solicitation, and eggshells and trash (e.g., plastic, food wrappers) were identified by sight. These methods are also reiterated in Hess et al. (2024).

### 2.5 Diet Estimation

We quantified coyote diets by visually assessing the percent composition that each diet item contributed to coyote scats (hereon referred to as volumetric percentage; Klare et al. 2011). This approach is a common method used to estimate the diets of wild animals (Capitani et al. 2004; Forman 2005; Thompson et al. 2022), particularly given the limited availability of fecal correction factors for many species (Klare et al. 2011), including coyotes (limitations of this approach are further elaborated upon in the Section 5). Diet remains that contributed to < 1% of the volume of a dried scat were not considered in analyses, as these items were expected to be incidental. Scats predominantly containing trash (> 50% volume), as well as eggshells—a minor component of two scats—were excluded from our analyses, due to an inability to accurately estimate the contents and subsequent macronutrient compositions of these diet items. Trash was also excluded due to an inability to identify to what degree the contents of these items were consumed. Similarly, scats whose volume was > 50% grass were excluded, as canids cannot digest grasses. Scats that had ≤ 50% grass or trash in them were included in our analyses, and these scats were assessed volumetrically while excluding these diet items.

After doing this across all scats individually, we then assessed the overall percent volume each diet item contributed collectively to scats across all sites, in low human influence sites, and in moderate human influence sites, and then in each of these categorizations by season. Thus, the diet composition of a scat represents a “meal” consumed by an individual animal, and the overall percent volume of all meals characterizes their diet. These data were then associated with estimates of the percent metabolizable energy (kcal) found within each diet item to determine the macronutrient composition of coyote diets, as described below.

### 2.6 Macronutrient Estimation

The total percent of metabolizable protein, carbohydrate, and lipid consumed was first determined relative to the percent volumetric contribution of those items to coyote diets and then summed across diet items to determine the overall proportion each macronutrient contributed to coyote diets within each stratum and by season. Specifically, macronutrient estimates for foods found in coyote scats were summarized from the literature and using methods derived from Coogan et al. (2014), Coogan, Raubenheimer, Stenhouse, et al. (2018). We identified studies that reported the percent of crude protein or lean dry mass, fat, and carbohydrate for whole carcasses of prey or for 100 g of vegetative matter. We then calculated the percent metabolizable energy of protein, lipids, and carbohydrates within each diet item using Atwater factors for the number of calories provided by each macronutrient per gram (Merrill and Watt 1973). For prey taxa, whole‐carcass estimates were used, as coyotes in our study system frequently preyed upon whole animals (e.g., small mammals), as evidenced by teeth, claws, and full skeletal structures of prey found in scats. However, coyotes also engage in scavenging behavior, particularly of larger‐bodied ungulate species (Gese et al. 1988; Pierce et al. 2000), and likely did not consume these animals as whole carcasses. The macronutrient composition of scavenged meats can vary substantially, primarily due to differences in the macronutrient composition of organ meat relative to flesh (Kohl et al. 2015). Likewise, animals often prey selectively on certain body parts when they have the opportunity (Kohl et al. 2015). Nonetheless, we used whole‐carcass estimates of ungulates, since it is likely coyotes consumed both organ meat and flesh while scavenging (Kohl et al. 2015), the latter of which was evidenced by the abundant ungulate hair found in coyote scats. Finally, for vegetation found in scats in the form of seeds, the pericarp or fruit associated with the plant was used for macronutrient estimates, as the presence of whole, intact seeds indicates fruit consumption.

Since the macronutrient estimates of food items can vary significantly depending upon season, environmental conditions, and the age of the animal (Kohl et al. 2015), we averaged multiple estimates across the literature or used references that reported values derived from larger sample sizes, when possible. When we could not identify estimates from the literature for a given species consumed by coyotes in our study system, we used a closely related species of similar body mass to derive estimates (Appendix S1: Tables S1 and S2). To determine the percent contribution of each macronutrient to coyote diets overall, as a function of the degree of urbanization, and within each season, we then multiplied the percent volume of scats containing each diet item (Appendix S1: Tables S3–S5) by the percent metabolizable energy calculated for each diet item (Appendix S1: Table S2). All percentages are reported as whole numbers.

### 2.7 Nutritional Geometry

We used right‐angled mixture triangles (RMTs) and the geometric framework of nutrition to explore the relationship between macronutrient consumption, season, and degree of urbanization (Raubenheimer 2011). RMTs visualize protein, fat, and carbohydrate consumption as percentages in multidimensional nutrient space (Raubenheimer 2011). Nutritional geometric and RMT analyses are visual, relational, and exploratory in nature and are often interpreted without formal statistical quantifications (Coogan, Raubenheimer, Zantis, and Machovsky‐Capuska 2018). Nonetheless, these methods have been used to assess the macronutrient compositions of several mammal species (Shrestha et al. 2020; Hecker et al. 2021), including other seasonally omnivorous carnivores (Coogan et al. 2014; Panthi et al. 2019). Furthermore, the geometric framework of nutrition applied in this study is a robust, established, and widely used method for visualizing and comparing the composition and regulation of macronutrients in animal diets, with applications in ecology, evolution, agriculture, wildlife management, and medicine (Simpson and Raubenheimer 2005, 2012; Raubenheimer et al. 2009, 2016; Jensen et al. 2012; Solon‐Biet et al. 2014; Coogan, Raubenheimer, Stenhouse, et al. 2018).

RMTs represent metabolizable energy in geometric space across three axes of variation (protein, carbohydrate, and lipid) (Raubenheimer 2011). Since the total percent energy provided across all macronutrients must equal 100%, the *z*‐axis (e.g., percent metabolizable energy from protein) varies relative to the proportion of macronutrients on the *x*‐ (e.g., percent metabolizable energy from carbohydrates) and *y*‐ (e.g., percent metabolizable energy from lipids) axes (Raubenheimer 2011). To visualize wild coyote diets relative to an intake target, we also included the intake target of domestic dogs (30% protein: 7% carbohydrate: 63% lipid; Hewson‐Hughes et al. 2013) on our RMTs. Though domestic dog intake targets likely differ from coyotes due to the associated effects of domestication on macronutrient selection (Hewson‐Hughes et al. 2013), the domestic dog intake target allows for the interpretation of results relative to what is currently known about macronutrient selection in a closely related *Canis* species (Lindblad‐Toh et al. 2005). In addition, current recommendations for captive coyote diets reflect those of domestic dogs (AZA Canid Taxon Advisory Group 2012), and, similar to coyotes, domestic dogs have an omnivorous diet (Arendt et al. 2014; Bosch et al. 2015). Finally, we used convex hull polygons to visualize differences in overall macronutrient space occupied by coyotes per season and degree of urbanization. Due to the nature of our analyses, the results presented are descriptive and do not have associated statistical values unless otherwise noted. All analyses were conducted in R version 4.1.2 (R Core Team 2021).

## 3 Results

### 3.1 Prey Consumption

**FIGURE 2:**
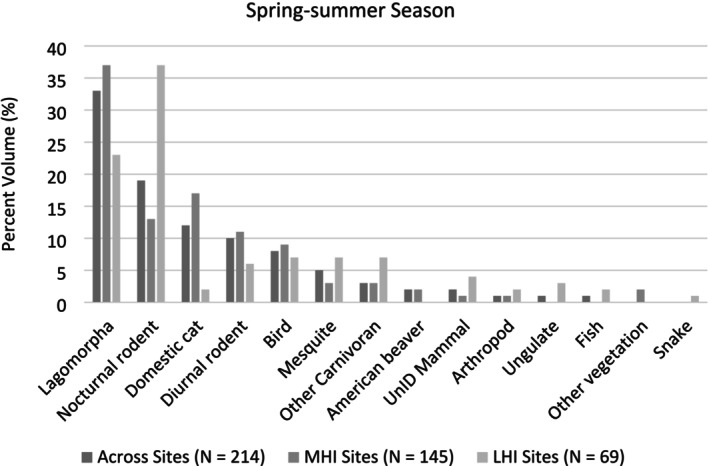
Summary of coyote diet occurrence in the spring–summer season, based on scat contents along the Salt River in the Phoenix Metropolitan Area, AZ, USA in low human influence (LHI) and moderate human influence (MHI) sites in the spring–summer season. The percent volume of diet items (%) in the collected scats are reported, and data are organized from the most to least frequently occurring diet items across sites. The percent volume of scat contents represented by each diet item are reported relative to all the scat analyzed within each grouping (i.e., across sites, MHI sites, or LHI sites). UnID refers to species that were unidentifiable.

**FIGURE 3:**
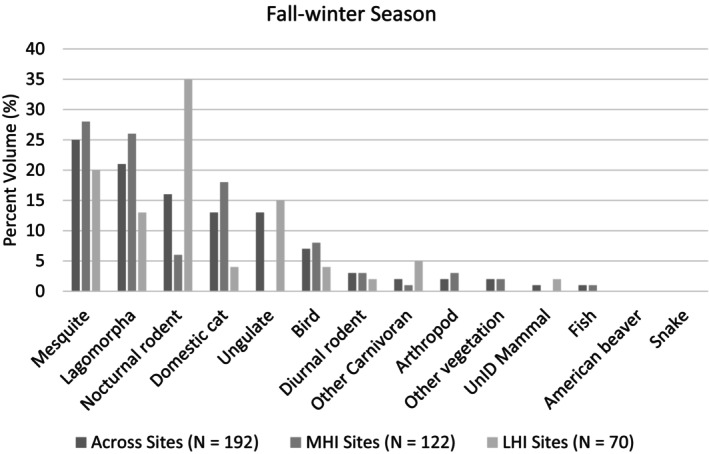
Summary of coyote diet occurrence in the fall–winter season, based on scat contents along the Salt River in the Phoenix Metropolitan Area, AZ, USA in low human influence (LHI) and moderate human influence (MHI) sites in the spring–summer season. The percent volume of diet items (%) in the collected scats are reported and data are organized from the most to least frequently occurring diet items across sites. The percent volume of scat contents represented by each diet item are reported relative to all the scat analyzed within each grouping (i.e., across sites, MHI sites, or LHI sites). UnID refers to species that were unidentifiable.

A total of 474 scats were collected, though 68 were excluded from analysis due to either uncertainty in identification, confirmation of not belonging to a coyote, or the sample mostly containing diet items we could not measure volumetrically, resulting in a total of 406 coyote scats being assessed overall (spring–summer: MHI = 145 scats, LHI = 69 scats; fall–winter: MHI = 122 scats, LHI = 70 scats, Appendix S1: Tables S3 and S4). Cottontail rabbits and small nocturnal rodents were the most frequent prey species found across sites and seasons in coyote scats, and domestic cats were also common contributors to coyote diets in MHI sites across seasons (Figures 2 and 3; Appendix S1: Tables S3 and S4). Coyote diets varied seasonally, with the greatest difference seen in the increased consumption of mesquite (*Prosopis* spp.) across sites in the fall–winter season and ungulate species in LHI sites in the fall–winter season (Figures 2 and 3; Appendix S1: Tables S3 and S4).

### 3.2 Macronutrient Composition of Diet Items

**FIGURE 4:**
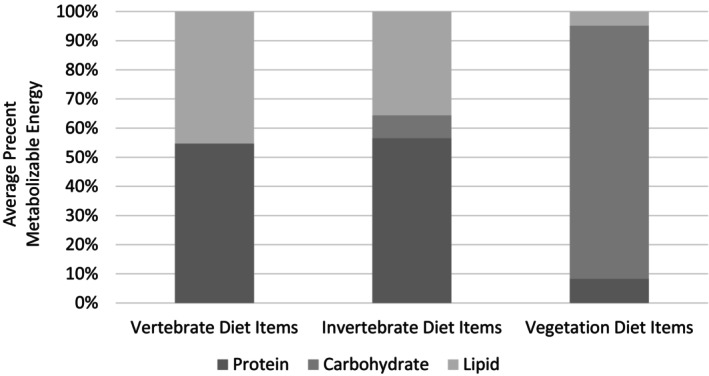
Average percent metabolizable energy derived from protein, carbohydrate, and lipid in assessed vertebrate, invertebrate, and vegetative diet items, as derived from the literature (see Appendix S1: Table S2).

The diet items consumed by coyotes, overall, mostly contained metabolizable energy from protein ($\bar{x}$: 48% [SD 19%]), and fat ($\bar{x}$: 38% [SD 16%]), with lower proportions of carbohydrates ($\bar{x}$: 13% [SD 31%]) (Appendix S1: Table S2). Much of the variation in these estimates can be attributed to vegetative matter, which was consistently higher in carbohydrate content compared with other food sources (Figure 4, Appendix S1: Table S2). Coyotes primarily consumed vertebrate species (Appendix S1: Tables S3 and S4), whose percent metabolizable energy from lipid and protein ranged from 42% to 73% protein ($\bar{x}$: 55% [SD 9%]) and 27%–58% fat ($\bar{x}$: 45% [SD 9%]) (Appendix S1: Table: S2).

### 3.3 Macronutrient Consumption in the Spring–Summer Season

Vertebrates contributed the most energy to coyote diets in the spring–summer season, with 55% of protein energy and 38% of lipid energy originating from vertebrate prey overall, with similar relationships observed in MHI and LHI sites individually (Appendix S1: Table S3). However, lagomorphs were a larger contributor to protein (22%) and fat (15%) energy in MHI sites, while nocturnal rodents were a larger source of energy from protein (24%) and fat (14%) in LHI sites (Appendix S1: Table S3). Vegetative matter and arthropods contributed minimally to coyote diets in the spring–summer season (Appendix S1: Table S3).

**FIGURE 5:**
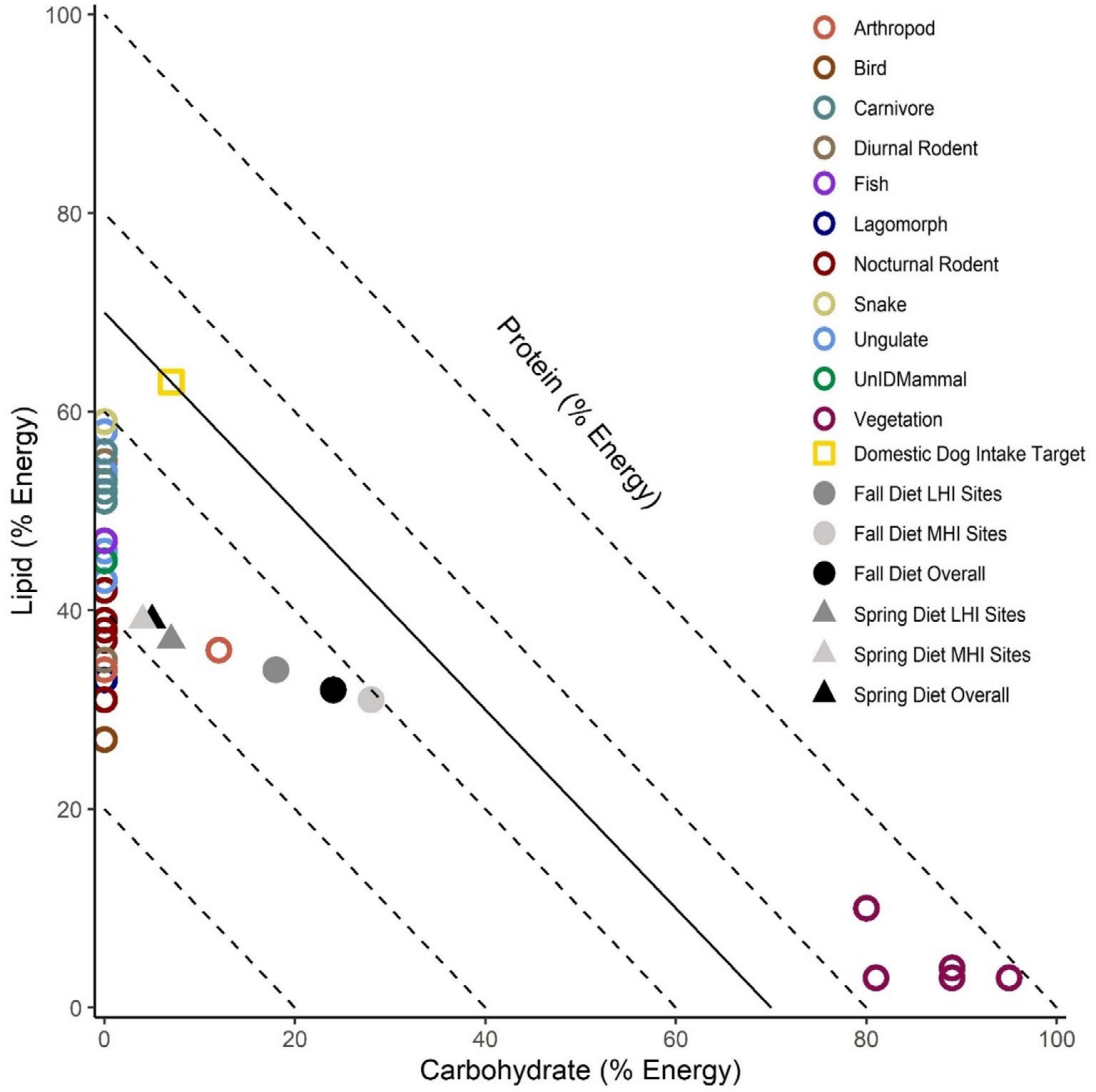
Right‐angled mixture triangle representing the macronutrient composition of diet items (empty circles), coyote diets overall (black points), in moderate human influence sites (MHI, light gray), in low human influence sites (dark gray), and by season (spring–summer: Colored triangles; fall–winter: Colored circles) in macronutrient space. The intake target of domestic dogs (Hewson‐Hughes et al. 2013) is represented as a reference point by an empty, yellow square and the solid black line, which indicates the ratio of protein to nonprotein energy associated with their intake target. When the macronutrient composition of coyote diets approaches this line, one can then interpret the results as suggesting coyotes consumed a similar ratio of protein compared to nonprotein energy relative to a reference point for canid species. Dashed lines represent the *z*‐axis, or percent metabolizable energy from protein at 0% (longest dashed line) to 80% (shortest dashed line) moving from the outside of the figure to the inside of the figure. Associated coyote scat data were collected along the Salt River corridor in the Phoenix Metropolitan Area, AZ, USA.

**FIGURE 6:**
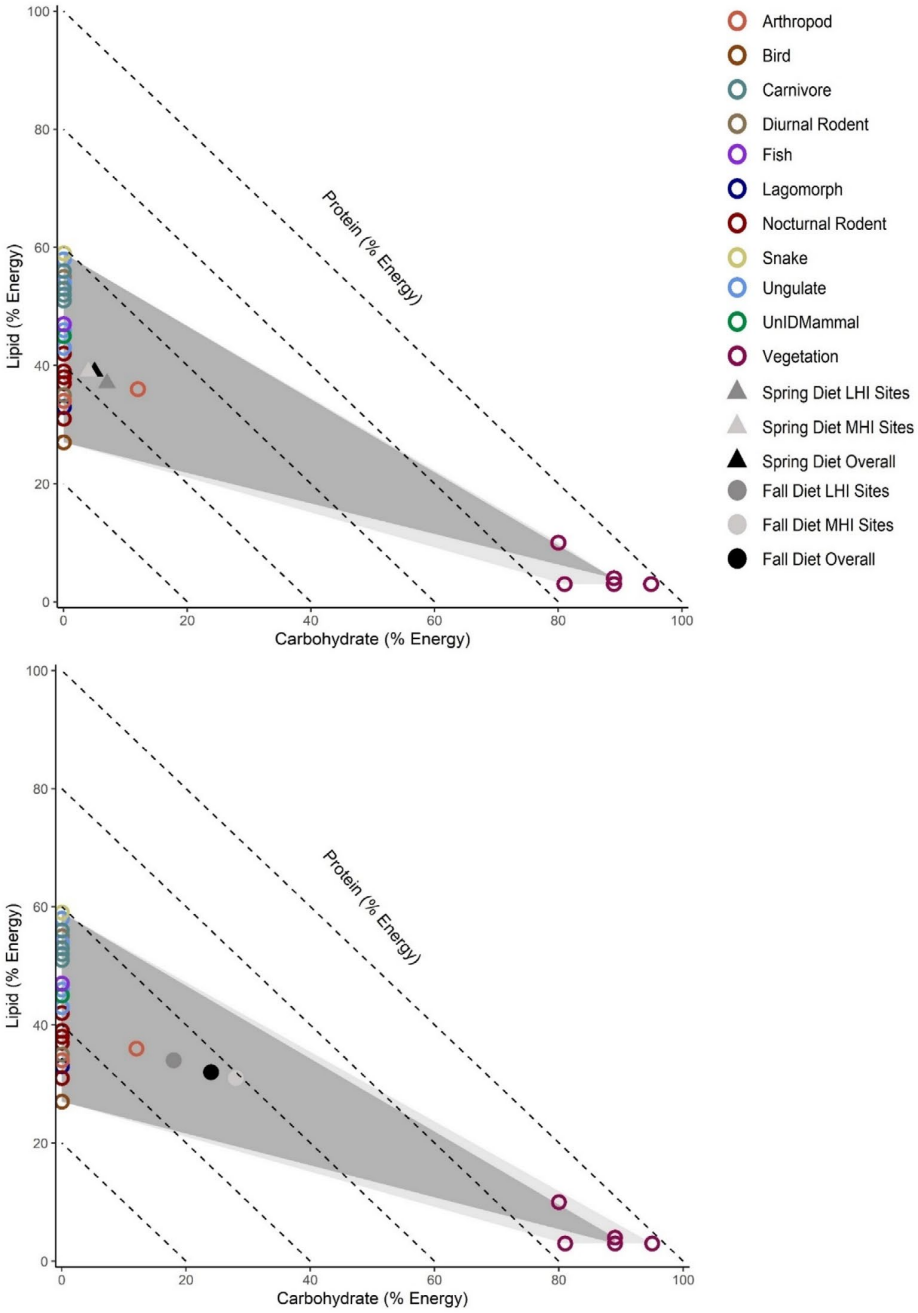
Convex hulls illustrating consumption in macronutrient space in in moderate human influence sites (MHI, light gray polygon) and in low human influence sites (dark gray polygon) in the spring–summer (triangles) and fall–winter (circles) seasons. Data are presented in right‐angled mixture triangles representing the macronutrient composition of diet items (empty circles), coyote diets overall (black triangle and circle), in MHI sites (light gray triangle and circle), and in LHI sites (dark gray triangle and circle) in macronutrient space. Dashed lines represent the *z*‐axis, or percent metabolizable energy from protein at 0% (longest dashed line) to 80% (shortest dashed line) moving from the outside of the figure to the inside of the figure. Associated coyote scat data were collected along the Salt River corridor in the Phoenix Metropolitan Area, AZ, USA.

RMT analysis indicated that coyote diets overall and in both low and moderate human influence sites were similar in terms of their macronutrient compositions in the spring–summer season. When comparing the percent metabolizable energy derived from protein relative to carbohydrate and lipid (i.e., P%:C%:L%) and protein to nonprotein energy ratios (%P:%NP) in the diets of coyotes, macronutrient compositions were 56:5:39 (56P:44NP) overall, 57:4:39 (57P:43NP) in MHI sites, and 56:7:37 (56P:44NP) in LHI sites (Figure 5; Appendix S1: Table S3). In terms of macronutrient space, convex hulls indicated diet items consumed by coyotes overlapped extensively, with a few more carbohydrate‐rich diet items eaten in moderate human influence sites (Figure 6).

### 3.4 Macronutrient Consumption in the Fall–Winter Season

Overall, vertebrates, predominately lagomorphs and nocturnal rodents, contributed the most energy to coyote diets, with 41% of protein energy and 30% of lipid energy originating from vertebrate prey in the fall–winter (Appendix S1: Table S4). However, unlike in the spring–summer, of the total 56% of nonprotein energy consumed by coyotes in the fall–winter season, 24% originated from vegetative matter and in the form of carbohydrates, largely sourced from mesquite (Appendix S1: Table S4). Coyotes in LHI sites derived more energy from vertebrate prey than in MHI sites, with coyotes receiving 46% (compared to 38% in MHI sites) of their protein energy and 33% (compared to 28% in MHI sites) of their lipid energy from vertebrates and 18% (compared to 27% in MHI sites) of their carbohydrate energy from vegetation in LHI sites (Appendix S1: Table S4).

RMT analysis indicated that coyote macronutrient consumption differed minimally between low and moderate human influence sites in the fall–winter season, with the introduction of carbohydrate‐rich foods (Figure 4). When comparing the percent protein to percent carbohydrate to percent lipid (i.e., P%:C%:L%) and protein to nonprotein energy ratios (%P:%NP) consumed by coyotes, coyote macronutrient compositions were 44:24:32 (44P:56NP) overall, 42:28:31 (42P:59NP) in moderate human influence sites, and 48:18:34 (48P:52NP) in low human influence sites (Figure 5; Appendix S1: Table S4). In terms of macronutrient space, convex hulls indicated diet items consumed by coyotes overlapped extensively, with more carbohydrate‐rich diet items eaten in moderate human influence sites (Figure 6).

## 4 Discussion

Our study is the first to summarize the macronutrient intake (i.e., of proteins, carbohydrates, and lipids) by coyotes generally, as well as relative to urbanization and seasonality. In line with our hypotheses, coyote macronutrient consumption changed with season and urbanization, though the latter was only the case in the fall–winter. Observed similarities in coyote macronutrient consumption between MHI and LHI sites in the spring–summer season may indicate a consistent macronutrient preference among animals, given the foods available in their respective environments. Alternatively, these similarities may result from the macronutrient content of available food items being comparable between locations. The available macronutrient niches were also similar in both the spring–summer and fall–winter seasons. However, it is also important to note that the convex hulls depicting the macronutrient niches available to the subpopulations do not show the relative abundance of different food items, which would have implications for the realized macronutrient niches and the overall balance of the coyotes' diets.

If we assume that the protein needs of coyotes in our study system were consistent and adequately met across seasons (i.e., that coyote protein requirements reflect those of domestic dogs, with protein contributing to a minimum of 30% of the animal's diet: Hewson‐Hughes et al. 2013), our results could indicate that coyotes foraged for particular macronutrients. This is evidenced by the increased percentage of nonprotein energy consumed in the fall–winter season compared with the spring–summer, particularly relative to the consistently high consumption of protein when compared to the reference domestic dog intake target (Hewson‐Hughes et al. 2013). This conclusion is further supported by the movement of macronutrient ratios towards those self‐selected by the closely related domestic dog‐fed ad libitum (i.e., toward the domestic dog intake target for P:NP energy) (Hewson‐Hughes et al. 2013). The observed increase in nonprotein energy relative to protein energy consumed by coyotes in the fall–winter could also indicate seasonal regulation of protein intake. Though it is unclear if domestic dog macronutrient needs fully reflect those of coyotes, the domestic dog intake target for protein is similar to that of other caniform species (e.g., bears, mink) (Erlenbach et al. 2014). As such, it is likely that the protein intake target of coyotes is similar to that of domestic dogs and other closely related species. Nonetheless, to truly determine whether coyote diets increased in nonprotein energy in the fall–winter due to protein regulation and not other drivers (e.g., interannual variability, individual preference, random chance, food availability, or social cues) one would need to conduct a repeated measures assessment of coyote diets and nutritional physiology across multiple years.

Differences in macronutrient consumption by coyotes by season, particularly of carbohydrates, could also be due to the seasonal availability of foods and/or coyote reproductive physiology. Similar to eastern coyotes, who have been observed to consume more vegetative matter based on the seasonal availability of plant species (Schrecengost et al. 2008; Swingen et al. 2015; Cherry et al. 2016), coyotes in our study system consumed large quantities of mesquite in the fall–winter season. Mesquite trees drop their carbohydrate‐rich (Harden and Zolfaghari 1988) seed pods in the fall (Ansley et al. 2017) and are abundantly available throughout the PMA (Martin et al. 2004). Furthermore, anthropogenic regions of the PMA experience greater primary productivity than wildland areas in drier years, due to water supplementation (Buyantuyev and Wu 2009). Thus, since our study was conducted in a drier year (NOAA 2021, 2022), mesquite trees in MHI sites may have produced more seed pods compared to LHI sites during our study period, resulting in their increased presence in coyote diets in moderately urbanized sites in the fall–winter. However, additional investigation of mesquite seed pod yields relative to urbanization, water supplementation, and weather patterns in the PMA is needed. Prior evidence also suggests that coyotes consume large quantities of mesquite after rearing pups and before the breeding season begins (i.e., in the fall–winter season) across rural and suburban contexts in other regions of Arizona (McClure et al. 1995). It is therefore possible that the timing of mesquite availability relative to coyote reproduction plays a role in coyotes' consumption of nonprotein energy in the fall–winter season, and not protein regulation. Due to its apparent importance in coyote diets in this region, future studies could tease out these dynamics using a combination of nutritional geometry and mesquite availability assessments relative to coyote reproductive phenology, particularly across multiple years and when comparing across sexes.

Lipid energy contributed a slightly smaller percentage to coyote diets in the fall–winter season compared with the spring–summer, but coyotes in both the fall and spring ate high amounts of fat‐rich foods, primarily in the form of domestic cats (both seasons in MHI sites) and ungulates (fall–winter season in LHI sites). The P:NP energy balance of coyote diets is important to consider because some animals will regulate to a P:NP ratio if their preferred ratio of macronutrient energy is unobtainable. For example, grizzly bears prefer diets with a high lipid content, but in its absence will consume more carbohydrate energy in order to maintain a preferred P:NP balance (Coogan et al. 2014). As a result, understanding under what conditions coyotes selectively forage for different macronutrients could support management efforts, for example, to better understand when coyotes consume foods high in carbohydrates (e.g., trash, seeds) compared to foods higher in lipids (e.g., domestic pets).

## 5 Limitations

Though this work presents the first full assessment of coyote macronutrient consumption, it also has several limitations. First and foremost, the analyses shown herein are descriptive and observational, due to small sample sizes. Current approaches determine the macronutrient composition of diets from scat contents at the aggregate level (e.g., Coogan et al. 2014; Coogan, Raubenheimer, Stenhouse, et al. 2018; Panthi et al. 2019; Hecker et al. 2021), limiting the application of statistical analyses. Future works that can determine the macronutrient composition of nonhuman animal diets from individual scats would increase the applicability of these approaches, as well as the degree of statistical inference possible when assessing individual study areas. Furthermore, determining if the results found herein are indicative of nutritionally driven diet selection and not other factors (e.g., random chance, interannual availability, and variability of diet items, as well as environmental constraints) requires repeated measures of individual animals and environmental conditions over the course of multiple years. We hope our project illustrates the need for such studies in the future.

Another limitation of our study was our inability to incorporate human‐associated foods in our analyses. Though trash remains in scats indicate that coyotes also consumed carbohydrate‐rich human foods in our study, trash, and other anthropogenic foods were excluded from our analyses due to an inability to quantify fully digested material (i.e., processed human foods that leave no discernable trace in scats). It is likely that the overall macronutrient availability and energy balance of coyotes would include a much higher proportion of carbohydrates within strata, particularly in MHI sites, and between seasons if human foods were quantified (e.g., Newsome et al. 2015; Murray, Cembrowski, et al. 2015; Larson et al. 2020; Jensen et al. 2022). As such, our results provide a conservative estimate of carbohydrate consumption by coyotes in the PMA across degrees of urbanization and season. Nonetheless, the frequency of coyote scats containing trash was relatively consistent between strata in the spring–summer and low in the fall–winter (spring–summer: 12% overall, 12% MHI sites, 14% LHI sites; fall–winter: 6% overall, 8% MHI sites, 3% LHI sites; Weiss 2024). It is therefore likely that any additional carbohydrate energy consumed from human foods was also consistent across strata (spring–summer) or negligible (fall–winter). Future studies using metabarcoding or related techniques (e.g., Machovsky‐Capuska, Priddel, et al. 2016; Henger et al. 2022) could help to elucidate the contributions of anthropogenic foods to coyote diets. Similarly, we used hard‐parts analyses of scats to quantify coyote diets, which can be biased toward less digestible food items and larger‐bodied animals (Klare et al. 2011). Though we addressed some of these limitations in our methods, fecal correction factors, which to our knowledge have yet to be developed for coyotes, could be created in the future to help improve estimates of coyote diets based on scat analyses.

Our study was also limited by our inability to use fecal correction factors to estimate food consumption by coyotes. One of the best methods for approximating diet from scat analysis is by using species‐specific (or similar) biomass correction factors applied to percent volume estimates to account for the differential digestibility of food items (Hewitt and Robbins 1996; Klare et al. 2011). However, fecal correction factors have been developed for relatively few species, and, in their absence, percent volume estimates are considered to be a suitable alternative for diet characterization (Klare et al. 2011). One downside of this approach, however, is that percent volume estimates do not account for the differential digestibility of prey items and tend to overestimate the intake of less digestible foods (e.g., fibrous vegetation) and underestimate the intake of highly digestible foods (e.g., meat). This could be particularly influential in our study, where coyotes were seen to consume high quantities of vegetative matter in the fall–winter season. However, in our case, we could not find a suitable biomass model for foods consumed by coyotes (i.e., fecal correction factors), and as such we estimated coyote diet using the percent volume approach. More specifically, though biomass estimates exist for the digestibility of some species by coyotes (e.g., small rodents: Weaver and Hoffman 1979; Kelly and Garton 1997), we could not find fecal correction factors for any other foods coyotes ate in our study system. We also investigated using fecal correction factors for closely related species as a proxy (e.g., gray wolves, *Canis lupus* : Weaver 1993); however, these species were also limited in the foods for which biomass models have been created, and lacked information on key species consumed by coyotes (e.g., domestic cats, cottontail rabbits, mesquite seeds). While not ideal, using the percent volume of diet items absent of correction factors can still yield valuable insights into the nutritional ecology of species and is a widely used and accepted approach (Capitani et al. 2004; Forman 2005; Klare et al. 2011; Thompson et al. 2022). This is particularly true of species that largely consume their prey whole, such as coyotes—as evidenced by whole rodent skeletons, the skull, teeth, and feet of cottontail rabbits, cat teeth and claws, and related items found in many coyote scats. Nonetheless, the field would benefit from studies in zoos or other captive coyote populations assessing the digestibility of various foods consumed by coyotes and other predator species that have diverse diets, particularly in and near cities, to better determine diet selection in future studies.

To determine whether our results are truly indicative of macronutrient selection and not simply availability or chance, the macronutrient composition of diets must also be compared to a known intake target—or preference of macronutrient ratios—for coyotes (Simpson and Raubenheimer 2012). Though we interpreted coyote macronutrient consumption relative to the closely related and omnivorous domestic dog's intake target, which provides some inferential power, the process of domestication alters nutritional physiology (Hewson‐Hughes et al. 2013) and so limits comparative ability. Knowing the intake target of coyotes would also support the application of our results for wildlife management. For example, perhaps coyote intake targets would indicate preferences for fats over carbohydrates when freely available (e.g., Hewson‐Hughes et al. 2013; Coogan et al. 2014). In addition, it is possible that individual coyotes had preferences for certain foods, thereby driving overall relationships (e.g., as has been observed in coyotes in San Francisco, CA: Caspi et al. 2025). Detailed information on this subject could therefore provide managers with insight into avenues of intervention that address root causes of human–wildlife conflicts, such as the consumption of domestic pets or other human‐associated foods (Soulsbury and White 2015) or identifying particular individuals of conflict concern. This study therefore also provides evidence in support of conducting a controlled, laboratory assessment of the coyote intake target in the future. Such an investigation could help our understanding of macronutrient intake regulation and its impacts on the evolution, health, and behavior of coyotes and closely related canid species, particularly in the age of the Anthropocene.

## Author Contributions

**Katherine C. B. Weiss:** conceptualization (lead), data curation (lead), formal analysis (lead), funding acquisition (equal), investigation (lead), methodology (supporting), project administration (supporting), software (lead), supervision (supporting), visualization (lead), writing – original draft (lead), writing – review and editing (lead). **Sean C. P. Coogan:** conceptualization (supporting), formal analysis (supporting), methodology (lead), validation (lead), writing – original draft (supporting), writing – review and editing (equal). **Pierre Deviche:** conceptualization (supporting), methodology (supporting), project administration (supporting), supervision (supporting), validation (equal), writing – review and editing (equal). **Jesse S. Lewis:** conceptualization (supporting), funding acquisition (equal), investigation (supporting), project administration (supporting), resources (equal), supervision (supporting), writing – review and editing (equal). **Savage C. Hess:** investigation (equal), project administration (supporting), writing – review and editing (equal). **Jan Schipper:** conceptualization (supporting), project administration (supporting), supervision (lead), writing – review and editing (equal). **Eric G. Strauss:** conceptualization (supporting), project administration (supporting), supervision (supporting), writing – review and editing (equal). **Beckett Sterner:** conceptualization (supporting), formal analysis (supporting), funding acquisition (equal), methodology (supporting), project administration (supporting), resources (lead), supervision (lead), writing – original draft (supporting), writing – review and editing (equal).

## Conflicts of Interest

The authors declare no conflicts of interest.

## Supporting information


Appendix S1

## Data Availability

All data used for the analyses can be found in the Supporting Information of this manuscript.
