# Supplementary material for: Coyote (Canis latrans) Macronutrient Consumption and Diet Relative to Seasonality and Urbanization
Source: Ecol Evol. 2025 May 12;15(5):e71405. doi: 10.1002/ece3.71405 (PMC12069223; doi:10.1002/ece3.71405)
Supplement: Supplementary file 1 — Appendix S1 [file ECE3-15-e71405-s001.docx]

Appendix S.1

**Title:** Coyote (*Canis latrans*) macronutrient consumption and diet relative to seasonality and urbanization

Table S.1. The percent metabolizable energy from proteins, carbohydrates, and lipids estimated from whole carcasses of coyote prey species or 100g of vegetative matter as determined from the literature.

| Taxonomic Group | Latin Name | Common Name | N | %P | %C | %L | Reference | Notes |
| --- | --- | --- | --- | --- | --- | --- | --- | --- |
| Arthropod | *Acrididae* Spp. | Grasshopper spp. | Thousands | 63 | 7 | 30 | Skipper et al. 2020 | Average across grasshoppers of varying body size (<10mm, 10-20mm, and >20mm) |
| Arthropod | *Coleoptera* Spp. | Beetle spp. | Not specified | 45 | 2 | 52 | Xiaoming et al. 2010 |  |
| Arthropod | *Lepidoptera* Spp. | Caterpillar spp. | 1740 | 48 | 26 | 26 | Skipper et al. 2020 | Lepidopteran larvae |
| Arthropod | *Procambarus clarkii* | Louisiana crayfish | 9 | 66 | 0 | 34 | Xu et al. 2013 |  |
| Bird | *Passeriformes* Spp. | Passerine bird spp. | 4 | 73 | 0 | 27 | Kremen et al. 2013 | Average across finches (n = 3) and a warbler (n = 1) |
| Diurnal Rodent | *Castor canadensis* | American beaver | 40-44 | 57 | 0 | 43 | Soprovich 1996 | 40 used for the protein estimate, 44 used for the fat estimate |
| Domestic Pet | *Canis familiaris* | Domestic dog | 3 | 44 | 0 | 56 | Sheng and Huggins 1971 | Average of 3 beagles > 1 year old |
| Domestic Pet | *Felis catus* | Domestic cat | 21 | 48 | 0 | 52 | Hendriks et al. 1997 | 14 male, 6 female adults |
| Fish | *Oncorhynchus clarkii* | Cutthroat trout | Not specified | 65 | 0 | 35 | Pritchard and Robbins 1990 |  |
| Fish | *Oncorhynchus mykiss* | Rainbow trout | Across 532-538 studies | 42 | 0 | 58 | Dumas et al. 2007 | 532 studies used to estimate lipids, 538 studies used to estimate protein |
| Lagomorph | *Lepus californicus* | Black-tailed jackrabbit 1 | 3 | 89 | 0 | 11 | Dierenfeld et al. 2002 |  |
| Lagomorph | *Lepus californicus* | Black-tailed jackrabbit 2 | 5 | 57 | 0 | 43 | MacCracken and Hansen 1986 |  |
| Lagomorph | *Oryctolagus Spp.* | Domestic rabbit spp. | 2 | 66 | 0 | 34 | Oyarzun et al. 1995 |  |
| Lagomorph | *Sylvilagus nuttalli* | Mountain cottontail | 5 | 56 | 0 | 44 | MacCracken and Hansen 1986 |  |
| Reptile | *Nerodia rhombifer* | Diamondback watersnake | 20 | 41 | 0 | 59 | Secor and Nagy 2003 |  |
| Small Diurnal Rodent | *Neotamias minimus* | Least chipmunk | 5 | 53 | 0 | 47 | MacCracken and Hansen 1986 |  |
| Small  Diurnal Rodent | *Sciurus carolinensis* | Gray squirrel | 74 | 62 | 0 | 38 | Powers et al. 1989 |  |
| Small  Diurnal Rodent | *Spermophilus townsendii* | Towsend’s ground squirrel | 5 | 37 | 0 | 63 | MacCracken and Hansen 1986 |  |
| Small  Diurnal Rodent | *Thomomys bottae* | Botta’s pocket gopher | 3 | 53 | 0 | 47 | Kremen et al. 2013 |  |
| Small  Diurnal Rodent | *Urocitellus columbianus* | Columbian ground squirrel | Not specified | 68 | 0 | 32 | Pritchard and Robbins 1990 |  |
| Small  Nocturnal Rodent | *Neotoma floridana* | Eastern woodrat | 28 | 69 | 0 | 31 | Snyder 2001 | Average across males, females, and seasons |
| Small  Nocturnal Rodent | *Peromyscus leucopus* | White-footed mouse | Not specified | 59 | 0 | 41 | Powers et al. 1989 |  |
| Small  Nocturnal Rodent | *Peromyscus maniculatus* | American deer mouse | 5 | 52 | 0 | 48 | MacCracken and Hansen 1986 |  |
| Small  Nocturnal Rodent | *Peromyscus* Spp. | Deer mouse | 64 | 62 | 0 | 38 | Hayward 1965 | Average across 6 species and, within those species, across seasons |
| Small  Nocturnal Rodent | *Rattus* Spp. | Rat spp. 1 | 4 | 77 | 0 | 23 | Kremen et al. 2013 | Average across two Norway rats and two Roof rats |
| Small  Nocturnal Rodent | *Rattus* Spp. | Rat spp. 2 | 51 | 47 | 0 | 53 | Dierenfeld et al. 2002 | Adults or > 50 g |
| Ungulate | *Bos taurus* | Domestic cow | 204 | 58 | 0 | 42 | Steen and Kilpatrick 1995 | Average value for bulls, steers, and heifers fed ad libitum and 80% ad libitum |
| Ungulate | *Capra hircus* | Domestic goat 1 | 32 | 71 | 0 | 29 | Alkass et al. 2014 | Average across kids: 16 males and 15 females |
| Ungulate | *Capra hircus* | Domestic goat 2 | 20 | 43 | 0 | 57 | Lerch et al. 2021 | Dairy goats |
| Ungulate | *Odocoileus hemionus* | Mule deer | 8 | 46 | 0 | 54 | Torbit et al. 1985 | Average of four trials across 8 adult (5 female, 3 castrated male) mule deer of high (n = 3), medium (n = 2), and low (n = 3) food intake |
| Ungulate | *Sus domesticus* | Domestic pig | 32 | 42 | 0 | 58 | Dierenfeld et al. 2002 |  |
| Vegetation | *Opuntia ficus-indica* | Prickly pear | Not specified | 2 | 95 | 3 | Salim et al. 2009 | Fruit only |
| Vegetation | *Phoenix dactylifera* | Date palm | Not specified | 16 | 81 | 3 | Sadiq et al. 2013 | Fruit only |
| Vegetation | *Prosopis glandulosa* | Honey mesquite | Not specified | 6 | 89 | 4 | Harden and Zolfaghari 1988 | Pericarp only |
| Vegetation | *Zea mays* | Corn | Not specified | 10 | 80 | 10 | USDA 2019 |  |
| Wild  Carnivore | *Canis latrans* | Coyote | 27 | 49 | 0 | 51 | Huot et al. 1995 | Eastern coyotes |

Table S.2. The percent metabolizable energy from protein, carbohydrates, and lipids estimated for each diet item found in coyote scats collected along the Salt River in the Phoenix Metropolitan Area, AZ, USA using the data presented in Appendix C.1.

| Diet Item | Species Data Used from Appendix C.1 | %P | %C | %L |
| --- | --- | --- | --- | --- |
| Desert Cottontail Rabbit | Average of mountain cottontail and domestic rabbit | 61 | 0 | 39 |
| Unidentified Lagomorph | Average of mountain cottontail, domestic rabbit, black-tailed jackrabbit 1 and 2 | 67 | 0 | 33 |
| Arizona Cotton Rat | Eastern woodrat | 69 | 0 | 31 |
| Neotominae Spp. | Average of eastern woodrat and the average of all Peromyscus spp. | 63 | 0 | 37 |
| Pocket Mouse | Average of all Peromyscus spp. | 58 | 0 | 42 |
| Rat Spp. | Average of rat spp. 1 and rat spp. 2 | 62 | 0 | 38 |
| Unknown Small Mammal | Average of eastern woodrat, each Peromyscus Spp., rat spp. 1, and rat spp. 2 | 61 | 0 | 39 |
| Rock squirrel | Average of gray squirrel and Columbian ground squirrel | 65 | 0 | 35 |
| Sciurid Spp. | Average of Towsend’s ground squirrel and least chipmunk | 45 | 0 | 55 |
| Botta’s Pocket Gopher | Botta’s pocket gopher | 53 | 0 | 47 |
| American Beaver | American beaver | 57 | 0 | 43 |
| Mule Deer | Mule deer | 46 | 0 | 54 |
| Collared Peccary | Domestic pig | 42 | 0 | 58 |
| Domestic Goat | Average of domestic goat 1and 2 | 57 | 0 | 43 |
| Domestic Ungulate | Average of domestic goat 1 and 2, domestic cow | 57 | 0 | 43 |
| Unidentified Ungulate | Average of mule deer, domestic goat 1 and 2, domestic cow | 54 | 0 | 46 |
| Coyote | Coyote | 49 | 0 | 51 |
| Domestic dog | Domestic dog | 44 | 0 | 56 |
| Bobcat | Coyote | 49 | 0 | 51 |
| Domestic cat | Domestic cat | 48 | 0 | 52 |
| Raccoon | Coyote | 49 | 0 | 51 |
| Unidentified Carnivore | Average of coyote, domestic cat, and domestic dog | 47 | 0 | 53 |
| Unidentified Mammal | Average of coyote, mule deer, domestic cat, gray squirrel, least chipmunk, eastern woodrat, mountain cottontail, and the average of all Peromyscus spp.) | 55 | 0 | 45 |
| Birds | Passerine birds | 73 | 0 | 27 |
| Snake | Diamondback watersnake | 41 | 0 | 59 |
| Fish | Average of rainbow trout and cutthroat trout | 53 | 0 | 47 |
| Insect | Average of grasshoppers, caterpillars, and beetles | 52 | 12 | 36 |
| Crayfish | Louisiana crayfish | 66 | 0 | 34 |
| Scorpion | Average of grasshoppers, caterpillars, and beetles | 52 | 12 | 36 |
| Mesquite | Honey mesquite | 6 | 89 | 4 |
| *Arecaceae* Spp. | Date palm | 16 | 81 | 3 |
| *Opuntia* Spp. | Prickly pear | 2 | 95 | 3 |
| Corn | Corn | 10 | 80 | 10 |
| Unknown Seed | Average of honey mesquite, date palm, and prickly pear | 8 | 89 | 3 |

Table S.3. Summary of coyote diets in the spring-summer season, based on scat contents along the Salt River in the Phoenix Metropolitan Area in Low Human Influence (LHI) and Moderate Human Influence (MHI) sites. The number of scats collected containing a diet item (n) and the percent volume that item contributed to coyote diets (%vol) are reported, as well as the percent metabolizable protein (%P), carbohydrate (%C), and lipid (%L) derived from each diet item relative to the percent volume it contributed to coyote diets. All percentages are rounded to whole numbers.

|  | Across sites | | | | | MHI Sites | | | | | LHI Sites | | | | |  |
| --- | --- | --- | --- | --- | --- | --- | --- | --- | --- | --- | --- | --- | --- | --- | --- | --- |
| Diet items | Occurrences | | | | | Occurrences | | | | | Occurrences | | | | |  |
|  | n | %vol | %P | %C | %L | n | %vol | %P | %C | %L | n | %vol | %P | %C | %L | |
| Lagomorpha | 93 | 33 | 20 | 0 | 13 | 69 | 37 | 22 | 0 | 15 | 24 | 23 | 14 | 0 | 8 | |
| *Sylvilagus audubonii* | 86 | 31 | 19 | 0 | 12 | 64 | 35 | 21 | 0 | 14 | 22 | 22 | 13 | 0 | 8 | |
| *UnID* | 6 | 2 | 1 | 0 | 1 | 4 | 2 | 1 | 0 | 1 | 2 | 1 | 1 | 0 | 0 | |
| Nocturnal Rodents | 78 | 19 | 13 | 0 | 7 | 34 | 13 | 7 | 0 | 4 | 44 | 37 | 24 | 0 | 14 | |
| *Sigmodon arizonae* | 23 | 7 | 5 | 0 | 2 | 13 | 6 | 4 | 0 | 2 | 10 | 8 | 6 | 0 | 3 | |
| *Neotominae spp.* | 32 | 8 | 5 | 0 | 3 | 10 | 3 | 2 | 0 | 1 | 22 | 19 | 12 | 0 | 7 | |
| *Chaetodipus spp.* | 21 | 4 | 3 | 0 | 2 | 9 | 2 | 1 | 0 | 1 | 12 | 10 | 6 | 0 | 4 | |
| *Rattus spp.* | 1 | 0 | 0 | 0 | 0 | 1 | 1 | 0 | 0 | 0 | 0 | 0 | 0 | 0 | 0 | |
| *UnID* | 1 | 0 | 0 | 0 | 0 | 1 | 1 | 0 | 0 | 0 | 0 | 0 | 0 | 0 | 0 | |
| Diurnal Rodents | 35 | 10 | 5 | 0 | 3 | 28 | 11 | 6 | 0 | 6 | 7 | 6 | 3 | 0 | 3 | |
| *Otospermophilus* *variegatus* | 10 | 4 | 3 | 0 | 1 | 8 | 5 | 3 | 0 | 2 | 2 | 2 | 1 | 0 | 1 | |
| *UnID Sciurid spp.* | 12 | 3 | 1 | 0 | 1 | 10 | 3 | 1 | 0 | 2 | 2 | 2 | 1 | 0 | 1 | |
| *Thomomys bottae* | 13 | 3 | 1 | 0 | 1 | 10 | 3 | 2 | 0 | 2 | 3 | 2 | 1 | 0 | 1 | |
| Large Rodents | 4 | 2 | 1 | 0 | 1 | 4 | 2 | 1 | 0 | 1 | 0 | 0 | 0 | 0 | 0 | |
| *Castor canadensis* | 4 | 2 | 1 | 0 | 1 | 4 | 2 | 1 | 0 | 1 | 0 | 0 | 0 | 0 | 0 | |
| Ungulates | 4 | 1 | 0 | 0 | 0 | 1 | 0 | 0 | 0 | 0 | 3 | 3 | 2 | 0 | 2 | |
| *Odocoileus hemionus* | 1 | 0 | 0 | 0 | 0 | 0 | 0 | 0 | 0 | 0 | 1 | 1 | 1 | 0 | 1 | |
| *Dicotyles tajacu* | 0 | 0 | 0 | 0 | 0 | 0 | 0 | 0 | 0 | 0 | 0 | 0 | 0 | 0 | 0 | |
| *Capra hircus* | 2 | 1 | 0 | 0 | 0 | 0 | 0 | 0 | 0 | 0 | 2 | 2 | 1 | 0 | 1 | |
| *Domestic ungulate* | 0 | 0 | 0 | 0 | 0 | 0 | 0 | 0 | 0 | 0 | 0 | 0 | 0 | 0 | 0 | |
| *UnID* | 1 | 0 | 0 | 0 | 0 | 1 | 0 | 0 | 0 | 0 | 0 | 0 | 0 | 0 | 0 | |
| Carnivorans | 51 | 15 | 8 | 0 | 9 | 40 | 20 | 9 | 0 | 11 | 11 | 9 | 5 | 0 | 5 | |
| *Canis latrans* | 1 | 0 | 0 | 0 | 0 | 0 | 0 | 0 | 0 | 0 | 1 | 1 | 1 | 0 | 1 | |
| *Canis familiaris* | 3 | 1 | 1 | 0 | 1 | 0 | 0 | 0 | 0 | 0 | 3 | 4 | 2 | 0 | 2 | |
| *Lynx rufus* | 0 | 0 | 0 | 0 | 0 | 0 | 0 | 0 | 0 | 0 | 0 | 0 | 0 | 0 | 0 | |
| *Felis catus* | 38 | 12 | 6 | 0 | 7 | 34 | 17 | 8 | 0 | 9 | 4 | 2 | 1 | 0 | 1 | |
| *Procyon lotor* | 1 | 0 | 0 | 0 | 0 | 0 | 0 | 0 | 0 | 0 | 1 | 1 | 1 | 0 | 1 | |
| *UnID* | 7 | 2 | 1 | 0 | 1 | 6 | 3 | 1 | 0 | 2 | 1 | 1 | 0 | 0 | 0 | |
| UnID Mammal | 5 | 2 | 1 | 0 | 1 | 2 | 1 | 0 | 0 | 0 | 3 | 4 | 2 | 0 | 2 | |
| Birds | 49 | 8 | 6 | 0 | 2 | 32 | 9 | 6 | 0 | 2 | 17 | 7 | 5 | 0 | 2 | |
| Snakes | 4 | 0 | 0 | 0 | 0 | 1 | 0 | 0 | 0 | 0 | 3 | 1 | 0 | 0 | 0 | |
| Fish | 4 | 1 | 0 | 0 | 0 | 0 | 0 | 0 | 0 | 0 | 4 | 2 | 1 | 0 | 1 | |
| Arthropods | 17 | 1 | 0 | 0 | 0 | 10 | 1 | 0 | 0 | 0 | 7 | 2 | 1 | 0 | 0 | |
| *Insect* | 13 | 1 | 0 | 0 | 0 | 9 | 1 | 0 | 0 | 0 | 4 | 1 | 0 | 0 | 0 | |
| *Crayfish* | 3 | 0 | 0 | 0 | 0 | 1 | 0 | 0 | 0 | 0 | 2 | 1 | 1 | 0 | 0 | |
| *Scorpion spp.* | 1 | 0 | 0 | 0 | 0 | 0 | 0 | 0 | 0 | 0 | 1 | 0 | 0 | 0 | 0 | |
| Vegetation | 43 | 5 | 0 | 4 | 0 | 31 | 5 | 0 | 5 | 0 | 12 | 7 | 0 | 6 | 0 | |
| *Prosopis spp.* | 28 | 5 | 0 | 4 | 0 | 17 | 3 | 0 | 3 | 0 | 11 | 7 | 0 | 6 | 0 | |
| *Arecaceae spp.* | 1 | 0 | 0 | 0 | 0 | 1 | 0 | 0 | 0 | 0 | 0 | 0 | 0 | 0 | 0 | |
| *Opuntia spp.* | 0 | 0 | 0 | 0 | 0 | 0 | 0 | 0 | 0 | 0 | 0 | 0 | 0 | 0 | 0 | |
| *Zea mays* | 1 | 0 | 0 | 0 | 0 | 1 | 1 | 0 | 1 | 0 | 0 | 0 | 0 | 0 | 0 | |
| *Other Seeds* | 2 | 0 | 0 | 0 | 0 | 2 | 1 | 0 | 1 | 0 | 0 | 0 | 0 | 0 | 0 | |
| Total | 214 | 100 | 56 | 5 | 39 | 145 | 100 | 57 | 4 | 39 | 69 | 100 | 56 | 7 | 37 | |

Table S.4. Summary of coyote diets in the fall-winter season, based on scat contents along the Salt River in the Phoenix Metropolitan Area in Low Human Influence (LHI) and Moderate Human Influence (MHI) sites. The number of scats collected containing a diet item (n) and the percent volume that item contributed to coyote diets (%vol) are reported, as well as the percent metabolizable protein (%P), carbohydrate (%C), and lipid (%L) derived from each diet item relative to the percent volume it contributed to coyote diets. All percentages are rounded to whole numbers.

|  | Across sites | | | | | MHI Sites | | | | | LHI Sites | | | | |
| --- | --- | --- | --- | --- | --- | --- | --- | --- | --- | --- | --- | --- | --- | --- | --- |
| Diet items | Occurrences | | | | | Occurrences | | | | | Occurrences | | | | |
|  | n | %vol | %P | %C | %L | n | %vol | %P | %C | %L | n | %vol | %P | %C | %L |
| Lagomorpha | 53 | 21 | 13 | 0 | 15 | 40 | 26 | 16 | 0 | 10 | 13 | 13 | 8 | 0 | 5 |
| *Sylvilagus audubonii* | 50 | 20 | 12 | 0 | 8 | 38 | 25 | 15 | 0 | 10 | 12 | 12 | 7 | 0 | 5 |
| *UnID* | 3 | 1 | 1 | 0 | 0 | 2 | 1 | 1 | 0 | 0 | 1 | 1 | 1 | 0 | 0 |
| Nocturnal Rodents | 65 | 16 | 10 | 0 | 7 | 27 | 6 | 3 | 0 | 3 | 38 | 35 | 22 | 0 | 14 |
| *Sigmodon arizonae* | 13 | 3 | 2 | 0 | 1 | 8 | 2 | 1 | 0 | 1 | 5 | 6 | 4 | 0 | 2 |
| *Neotominae spp.* | 24 | 7 | 4 | 0 | 3 | 7 | 2 | 1 | 0 | 1 | 17 | 15 | 10 | 0 | 6 |
| *Chaetodipus spp.* | 27 | 6 | 4 | 0 | 3 | 12 | 2 | 1 | 0 | 1 | 15 | 14 | 8 | 0 | 6 |
| *Rattus spp.* | 0 | 0 | 0 | 0 | 0 | 0 | 0 | 0 | 0 | 0 | 0 | 0 | 0 | 0 | 0 |
| *UnID* | 1 | 0 | 0 | 0 | 0 | 0 | 0 | 0 | 0 | 0 | 1 | 0 | 0 | 0 | 0 |
| Diurnal Rodents | 13 | 3 | 1 | 0 | 1 | 10 | 3 | 2 | 0 | 2 | 3 | 2 | 1 | 0 | 1 |
| *Otospermophilus* *variegatus* | 0 | 0 | 0 | 0 | 0 | 0 | 0 | 0 | 0 | 0 | 0 | 0 | 0 | 0 | 0 |
| *UnID Sciurid spp.* | 9 | 2 | 1 | 0 | 1 | 6 | 2 | 1 | 0 | 1 | 3 | 2 | 1 | 0 | 1 |
| *Thomomys bottae* | 4 | 1 | 0 | 0 | 0 | 4 | 1 | 1 | 0 | 1 | 0 | 0 | 0 | 0 | 0 |
| Large Rodents | 0 | 0 | 0 | 0 | 0 | 0 | 0 | 0 | 0 | 0 | 0 | 0 | 0 | 0 | 0 |
| *Castor canadensis* | 0 | 0 | 0 | 0 | 0 | 0 | 0 | 0 | 0 | 0 | 0 | 0 | 0 | 0 | 0 |
| Ungulates | 16 | 13 | 3 | 0 | 4 | 3 | 0 | 1 | 0 | 1 | 13 | 15 | 7 | 0 | 7 |
| *Odocoileus hemionus* | 7 | 3 | 1 | 0 | 2 | 0 | 0 | 0 | 0 | 0 | 7 | 9 | 4 | 0 | 5 |
| *Dicotyles tajacu* | 3 | 1 | 1 | 0 | 1 | 3 | 2 | 1 | 0 | 1 | 0 | 0 | 0 | 0 | 0 |
| *Capra hircus* | 2 | 1 | 0 | 0 | 0 | 0 | 1 | 0 | 0 | 0 | 2 | 0 | 0 | 0 | 0 |
| *Domestic ungulate* | 4 | 2 | 1 | 0 | 1 | 0 | 0 | 0 | 0 | 0 | 4 | 6 | 3 | 0 | 2 |
| *UnID* | 0 | 0 | 0 | 0 | 0 | 0 | 0 | 0 | 0 | 0 | 0 | 0 | 0 | 0 | 0 |
| Carnivorans | 42 | 15 | 6 | 0 | 8 | 33 | 19 | 9 | 0 | 9 | 9 | 9 | 3 | 0 | 5 |
| *Canis latrans* | 2 | 0 | 0 | 0 | 0 | 0 | 0 | 0 | 0 | 0 | 2 | 1 | 0 | 0 | 1 |
| *Canis familiaris* | 4 | 1 | 0 | 0 | 1 | 2 | 1 | 0 | 0 | 0 | 2 | 1 | 0 | 0 | 1 |
| *Lynx rufus* | 2 | 1 | 0 | 0 | 0 | 0 | 0 | 0 | 0 | 0 | 2 | 3 | 1 | 0 | 1 |
| *Felis catus* | 34 | 13 | 6 | 0 | 7 | 31 | 18 | 9 | 0 | 9 | 3 | 4 | 2 | 0 | 2 |
| *Procyon lotor* | 0 | 0 | 0 | 0 | 0 | 0 | 0 | 0 | 0 | 0 | 0 | 0 | 0 | 0 | 0 |
| *UnID* | 0 | 0 | 0 | 0 | 0 | 0 | 0 | 0 | 0 | 0 | 0 | 0 | 0 | 0 | 0 |
| UnID Mammal | 2 | 1 | 1 | 0 | 1 | 1 | 0 | 0 | 0 | 0 | 1 | 2 | 1 | 0 | 1 |
| Birds | 31 | 7 | 5 | 0 | 2 | 21 | 8 | 6 | 0 | 2 | 10 | 4 | 3 | 0 | 1 |
| Snakes | 3 | 0 | 0 | 0 | 0 | 1 | 0 | 0 | 0 | 0 | 2 | 0 | 0 | 0 | 0 |
| Fish | 3 | 1 | 0 | 0 | 0 | 1 | 1 | 1 | 0 | 0 | 2 | 0 | 0 | 0 | 0 |
| Arthropods | 16 | 2 | 1 | 0 | 1 | 11 | 3 | 2 | 0 | 1 | 5 | 0 | 0 | 0 | 0 |
| *Insect* | 14 | 2 | 1 | 0 | 1 | 10 | 3 | 2 | 0 | 1 | 4 | 0 | 0 | 0 | 0 |
| *Crayfish* | 1 | 0 | 0 | 0 | 0 | 1 | 0 | 0 | 0 | 0 | 0 | 0 | 0 | 0 | 0 |
| *Scorpion spp.* | 0 | 0 | 0 | 0 | 0 | 0 | 0 | 0 | 0 | 0 | 0 | 0 | 0 | 0 | 0 |
| Vegetation | 80 | 27 | 2 | 24 | 1 | 59 | 30 | 2 | 27 | 1 | 21 | 20 | 1 | 18 | 1 |
| *Prosopis spp.* | 70 | 25 | 2 | 22 | 1 | 51 | 28 | 2 | 25 | 1 | 19 | 20 | 1 | 18 | 1 |
| *Arecaceae spp.* | 2 | 1 | 0 | 1 | 0 | 2 | 1 | 0 | 1 | 0 | 0 | 0 | 0 | 0 | 0 |
| *Opuntia spp.* | 3 | 1 | 0 | 1 | 0 | 3 | 1 | 0 | 1 | 0 | 0 | 0 | 0 | 0 | 0 |
| *Zea mays* | 2 | 0 | 0 | 0 | 0 | 1 | 0 | 0 | 0 | 0 | 1 | 0 | 0 | 0 | 0 |
| *Other Seeds* | 3 | 0 | 0 | 0 | 0 | 2 | 0 | 0 | 0 | 0 | 1 | 0 | 0 | 0 | 0 |
| Total | 192 | 100 | 44 | 24 | 32 | 122 | 100 | 42 | 28 | 31 | 70 | 100 | 48 | 18 | 34 |

Table S5. Examples of scat samples and their percent volume contributions per diet item.

| **Scat sample** | **Percent Volume Contributions** |
| --- | --- |
| 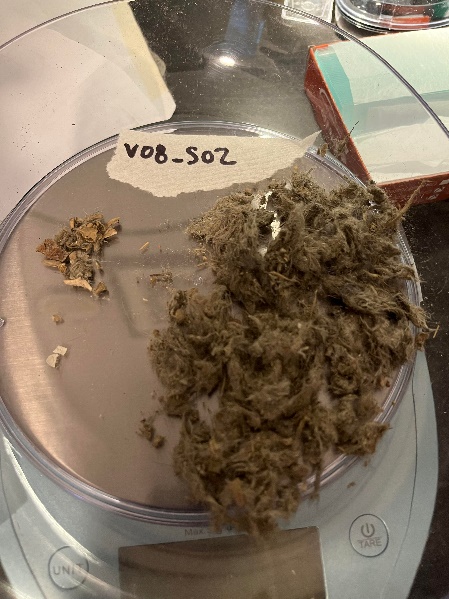 | 100% cottontail rabbit |
| 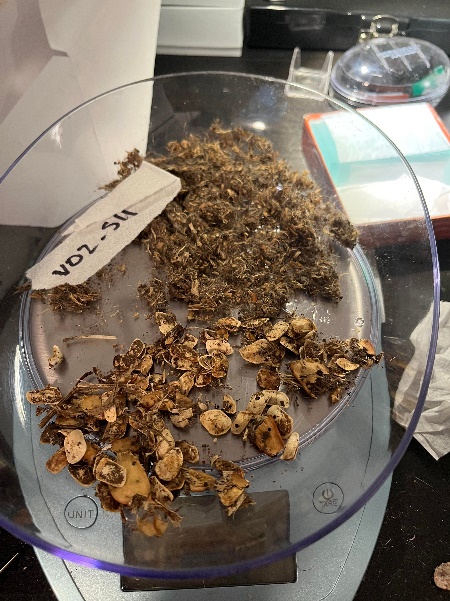 | 50% mesquite seeds; 50% Arizona cotton rat |
| 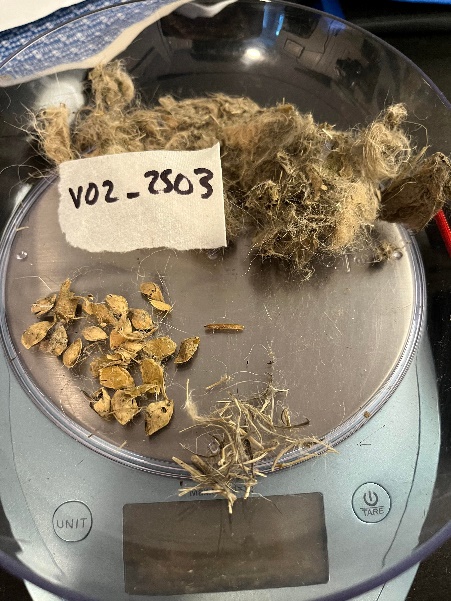 | 80% domestic cat; 10% bird; 10% mesquite seeds |

**References**

Alkass, J. E., K. A. D. Oray, and M. K. Abdulla. 2014. Studies on growth, carcass traits and body composition of goats raised either in intensive or pasture conditions (2- body composition and carcass tissue distribution). Advances in Life Science and Technology 19:15–22.

Dierenfeld, E. S., H. L. Alcorn, and K. L. Jacobsen. 2002. Nutrient composition of whole vertebrate prey (excluding fish) fed in zoos.

Dumas, A., C. F. M. De Lange, J. France, and D. P. Bureau. 2007. Quantitative description of body composition and rates of nutrient deposition in rainbow trout (*Oncorhynchus mykiss*). Aquaculture 273:165–181.

Harden, M. L., and R. Zolfaghari. 1988. Nutritive composition of green and ripe pods of honey mesquite (*Prosopis glandulosa*, Fabaceae). Economic Botany 42:522–532.

Hayward, J. S. 1965. The gross body composition of six geographic races of *Peromyscus*. Canadian Journal of Zoology 43:297–308.

Hendriks, W. H., P. J. Moughan, and M. F. Tarttelin. 1997. Body composition of the adult domestic cat (*Felis catus*). Journal of Animal Physiology and Animal Nutrition 77:16–23.

Huot, J., M.-L. Poulle, and M. Crête. 1995. Evaluation of several indices for assessment of coyote (*Canis latrans*) body composition. Canadian Journal of Zoology 73:1620–1624.

Kremen, N. A., C. C. Calvert, J. A. Larsen, R. A. Baldwin, T. P. Hahn, and A. J. Fascetti. 2013. Body composition and amino acid concentrations of select birds and mammals consumed by cats in northern and central California. Journal of Animal Science 91:1270–1276.

Lerch, S., A. De La Torre, C. Huau, M. Monziols, C. Xavier, L. Louis, Y. Le Cozler, P. Faverdin, P. Lamberton, I. Chery, D. Heimo, C. Loncke, P. Schmidely, and J. A. A. Pires. 2021. Estimation of dairy goat body composition: A direct calibration and comparison of eight methods. Methods 186:68–78.

MacCracken, J. G., and R. M. Hansen. 1986. Energy and protein content of coyote prey in southeastern Idaho. The Great Basin Naturalist 46:274–276.

Oyarzun, S. E., K. Self, E. V. Valdes, and E. R. Chavez. 1995. An evaluation of the nutritional adequacy of the feeding program of the black-footed ferret (*Mustela nigripis*) at the Metropolitan Toronto Zoo. Nutrition Advisory Group, Toronto.

Powers, J. G., W. W. Mautz, and P. J. Pekins. 1989. Nutrient and energy assimilation of prey by bobcats. The Journal of Wildlife Management 53:1004.

Pritchard, G. T., and C. T. Robbins. 1990. Digestive and metabolic efficiencies of grizzly and black bears. Canadian Journal of Zoology 68:1645–1651.

Sadiq, I. S., T. Izuagie, M. Shuaibu, A. I. Dogoyaro, A. Garba, and S. Abubakar. 2013. The nutritional evaluation and medicinal value of date palm (*Phoenix dactylifera*).

Salim, N., C. Abdelwaheb, C. Rabah, and B. Ahcene. 2009. Chemical composition of *Opuntia ficus-indica* (L.) fruit.

Secor, S. M., and T. R. Nagy. 2003. Non-invasive measure of body composition of snakes using dual-energy X-ray absorptiometry. Comparative Biochemistry and Physiology Part A: Molecular & Integrative Physiology 136:379–389.

Sheng, H. P., and R. A. Huggins. 1971. Growth of the beagle: Changes in chemical composition. Growth 35:369–376.

Skipper, B. R., D. Kim, and C. Morris. 2020. Seasonal abundance and nutritional concentration of grassland arthropods. Western North American Naturalist 80:19.

Snyder, M. V. 2001. Seasonal variation in hematology, body composition, and food caches of eastern woodrats (*Neotoma floridana*). Master of Science, Emporia State University.

Soprovich, D. W. 1996. Seasonal patterns of body composition and gut morphology of beaver (*Castor canadensis*). University of Manitoba, Ottawa.

Steen, R. W. J., and D. J. Kilpatrick. 1995. Effects of plane of nutrition and slaughter weight on the carcass composition of serially slaughtered bulls, steers and heifers of three breed crosses. Livestock Production Science 43:205–213.

Torbit, S. C., L. H. Carpenter, A. W. Alldredge, and D. M. Swift. 1985. Mule deer body composition: A comparison of methods. The Journal of Wildlife Management 49:86.

USDA. 2019. Corn grain, yellow. https://fdc.nal.usda.gov/fdc-app.html#/food-details/170288/nutrients.

Xiaoming, C., F. Ying, Z. Hong, and C. Zhiyong. 2010. Review of the nutritive value of edible insects. Pages 85–92 *in* P. B. Durst, D. V. Johnson, R. N. Leslie, and K. Shono, editors. Forest insects as food: humans bite back. Food and Agriculture Organization of the United Nations, Regional Office for Asia and the Pacific, Bangkok, Thailand.

Xu, W.-N., W.-B. Liu, M. Shen, G.-F. Li, Y. Wang, and W. Zhang. 2013. Effect of different dietary protein and lipid levels on growth performance, body composition of juvenile red swamp crayfish (*Procambarus clarkii*). Aquaculture International 21:687–697.
